# The Receptor Binding Domain of SARS-CoV-2 Lambda Variant Has a Better Chance Than the Delta Variant in Evading BNT162b2 COVID-19 mRNA Vaccine-Induced Humoral Immunity

**DOI:** 10.3390/ijms231911325

**Published:** 2022-09-26

**Authors:** Haolin Liu, Pengcheng Wei, Katja Aviszus, Qianqian Zhang, Jared Linderberger, John Yang, Junfeng Liu, Zhongzhou Chen, Hassan Waheed, Lyndon Reynoso, Gregory P. Downey, Stephen K. Frankel, John W. Kappler, Philippa Marrack, Gongyi Zhang

**Affiliations:** 1 Department of Immunology and Genomic Medicine, National Jewish Health, Denver, CO 80206, USA; 2 State Key Laboratory of Agrobiotechnology, College of Biological Sciences, China Agriculture University, Beijing 100193, China; 3 Department of Biochemistry and Molecular Genetics School of Medicine, Anschutz Medical Center, University of Colorado, Aurora, CO 80216, USA; 4 Department of Medicine, National Jewish Health, Denver, CO 80206, USA; 5 Department of Pharmacy, National Jewish Health, Denver, CO 80206, USA

**Keywords:** SARS-CoV-2, mutations, ACE2, Bamlanivimab, serum antibody

## Abstract

The SARS-CoV-2 Delta and Lambda variants had been named variants of concern (VOC) and variants of interest (VOI), respectively, by the World Health Organization (WHO). Both variants have two mutations in the spike receptor binding domain (RBD) region, with L452R and T478K mutations in the Delta variant, and L452Q and F490S mutations in the Lambda variant. We used surface plasmon resonance (SPR)-based technology to evaluate the effect of these mutations on human angiotensin-converting enzyme 2 (ACE2) and Bamlanivimab binding. The affinity for the RBD ligand, ACE2, of the Delta RBD is approximately twice as strong as that of the wild type RBD, an increase that accounts for the increased infectivity of the Delta variant. On the other hand, in spite of its amino acid changes, the Lambda RBD has similar affinity to ACE2 as the wild type RBD. The protective anti-wild type RBD antibody Bamlanivimab binds very poorly to the Delta RBD and not at all to the Lambda RBD. Nevertheless, serum antibodies from individuals immunized with the BNT162b2 vaccine were found to bind well to the Delta RBD, but less efficiently to the Lambda RBD in contrast. As a result, the blocking ability of ACE2 binding by serum antibodies was decreased more by the Lambda than the Delta RBD. Titers of sera from BNT162b2 mRNA vaccinated individuals dropped 3-fold within six months of vaccination regardless of whether the target RBD was wild type, Delta or Lambda. This may account partially for the fall off with time in the protective effect of vaccines against any variant.

## 1. Introduction

SARS-CoV-2 continues to cause millions of infections, hospitalizations and deaths throughout the world [1]. The virus infects via engagement of human ACE2 by the virus receptor binding domain (RBD) of its spike protein [2]. Although the RBD is only one fifth the length of the spike protein ectodomain, over 90% of broadly neutralizing antibodies from convalescent patients as well as vaccinated individuals engage the RBD [3,4]. Monoclonal antibodies (mAbs) specifically targeting the RBD have been approved by the FDA for emergency use [5]. However, the increasing number of SARS-CoV-2 variants, most with mutations in the RBD region, that have appeared around the world raises concerns about the efficacy of mAbs and serum antibodies raised by vaccines [6]. 

The SARS-CoV-2 Delta variant, a variant of concern (VOC), was the dominant strain worldwide before the Omicron variant [7]. Delta has two mutations within its RBD, L452R and T478K. The Lambda variant, once a major strain in Argentina and Chile, was named a variant of interest (VOI) [8]. Like Delta, Lambda has two mutations in its RBD, L452Q and F490S. Here we decipher the effect of these mutations on binding of ACE2 and the monoclonal antibody, Bamlanivimab. We also report that the level of anti-RBD antibodies, against wild type and the Delta and Lambda RBDs, falls by about 70% within six months of BNT162b2 mRNA COVID-19 vaccination encoding the wild type spike protein. Moreover, the Lambda RBD evades more serum antibody binding than Delta.

## 2. Results

### 2.1. The Delta RBD Has Increased ACE2 Binding Affinity by Two Fold, While the Lambda RBD Has Similar Affinity as the Wild Type

**Figure 1:**
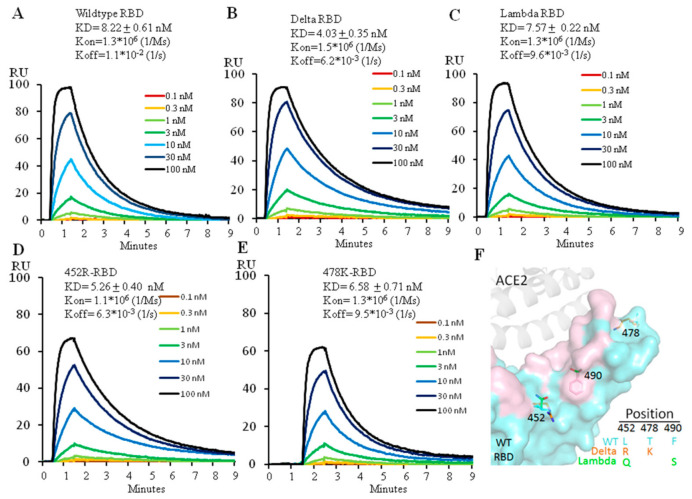
Affinity measurement of ACE2 binding to wild type (**A**), Delta (**B**), Lambda (**C**), 452R (**D**) or 478K (**E**) RBD using Biacore. The protein model for RBD and ACE2 is shown in (**F**) with the ACE2 footprint in pink. 6M0J (PDB ID) was used for modeling.

The two amino acid changes, L452R and T478K, distinguish the Delta RBD from its ancestor. To find out how each mutation affects the ability of the Delta versus wild type RBD to engage ACE2, we expressed the Delta RBD and its individual changes, L452R or T478K and the wild type RBD (Figure S1) and used Biacore to analyze their binding affinity for ACE2. The wild type RBD bound ACE2 at 8.2 nM (Figure 1A), a value similar to that previously reported [2]. The Delta variant RBD increased ACE2 affinity by twofold, at 4.0 nM (Figure 1B), consistent with the previous report [9]. Measurement of the effects of the individual amino acid changes showed that the affinities for ACE2 of L452R-RBD and T478K-RBD were 5.3 nM and 6.6 nM, respectively, indicating that the L452R mutation alone contributed more to the higher affinity to ACE2 than the T478K mutation alone (Figure 1C,D). The increase of ACE2 affinity by L452R mutation is due to the electrostatic interaction between the positively charged arginine side chain and the negatively charged patch on the ACE2 surface, reported by different groups [10,11,12]. However, the T478K mutation has been reported to form additional interactions with ACE2 in combination with L452R, leading to a higher affinity of the Delta RBD to ACE2 [9]. 

The Lambda RBD also has two mutations, L452Q and F490S. We expressed the Lambda RBD (452Q plus 490S) (Figure S1) and measured its binding affinity for ACE2 with Biacore. The affinity of the Lambda variant RBD for ACE2 was 7.6 nM (Figure 1E), similar to that of the wild type RBD. Neither L452Q nor F490S mutations individually affected ACE2 binding affinity (Unpublished data) as they don’t come into direct contact with ACE2 (Figure 1F). As the sidechain of glutamine is not positively charged, it therefore cannot form electrostatic interactions with ACE2 as arginine does in the Delta variant. 

### 2.2. The L452R Mutation in the Delta RBD Decreased Bamlanivimab Binding by 23-Fold. While Both Mutations in the Lambda Variant Collaborate to Abolish Bamlanivimab Binding

**Figure 2:**
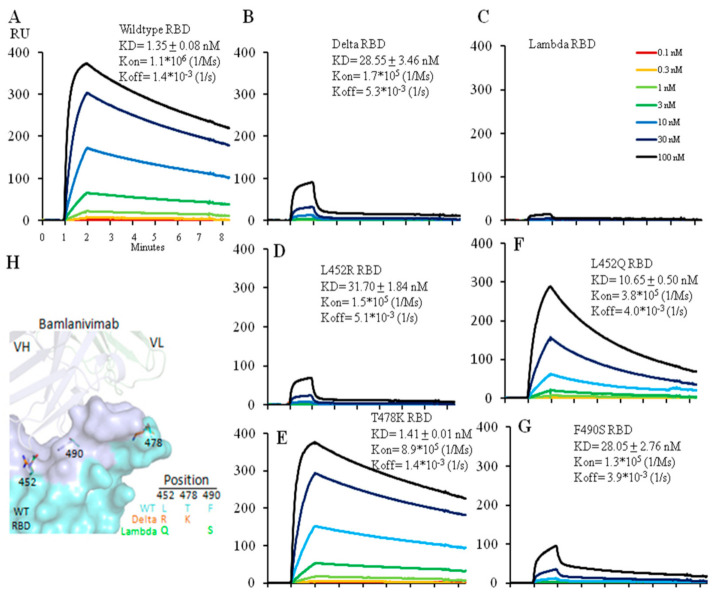
Affinity measurement of Bamlanivimab binding to wild type (**A**), Delta (**B**) or Lambda (**C**) RBD with Biacore. Bamlanivimab binding to RBDs with a single amino acid mutation was also measured to dissect each mutation’s contribution to Bamlanivimab binding with two Delta mutations L452R (**D**), T478K (**E**), and two Lambda mutations L452Q (**F**) and F490S (**G**). The protein model for Bamlanivimab and RBD is shown in (**H**) with the Bamlanivimab footprint on RBD in light blue while the other part of RBD in cyan. 7KMG (PDB ID) was used for modeling. Note that all the Biacore figures have the same x-axis unit as in the (**A**).

Via Biacore we tested the affinity of the neutralizing antibody, Bamlanivimab, for the wild type, Delta and Lambda RBDs. Bamlanivimab binds the wild type RBD with an affinity of 1.4 nM and the Delta RBD much less efficiently with an affinity of 28.5 nM. Of the two amino acid changes in the RBD between the wild type and Delta version, the L452R mutation alone accounted for the entire drop in affinity whereas the other, T478K mutation, had no effect (Figure 2A,B,D,E). L452 in the RBD is located within the hydrophobic interaction interface between the wild type RBD and Bamlanivimab (Figure 2H and Figure S2), thus the introduction of a positive charge by the L452R mutation completely disrupts the hydrophobic interface (Figure S2) [12], accounting for the dramatic drop in antibody affinity for the Delta RBD (Figure 2B,D). T478 does not lie in this interface, and therefore the T478K mutation had no impact on the antibody affinity (Figure 2E).

We carried out similar affinity measurements for the interaction between Bamlanivimab and the Lambda RBD. Bamlanivimab did not bind the Lambda RBD at all (Figure 2C). Analysis of the two mutations in the Lambda RBD separately showed that the L452Q mutation reduced the affinity of the interaction by ~8-fold (from 1.4 nM to 10.7 nM) (Figure 2F) presumably because, like the Delta L452R, the introduction of glutamine at this same site weakens the hydrophobic interactions between the RBD and Banlanivimab. The Lambda RBD F490S mutation lead to a ~20-fold decrease (from 1.4 nM to 28.0 nM) (Figure 2G) in the affinity of the interaction. F490 is located in the hydrophobic interface between the RBD and the antibody (Figure S2), and F490S mutation accounted for the fall in affinity. Thus, the two mutations, L452Q and F490S, accounted for the complete loss in antibody binding. 

### 2.3. Bamlanivimab Decreased and Lost Neutralization Efficacy against the Delta and the Lambda RBD, Respectively

**Figure 3:**
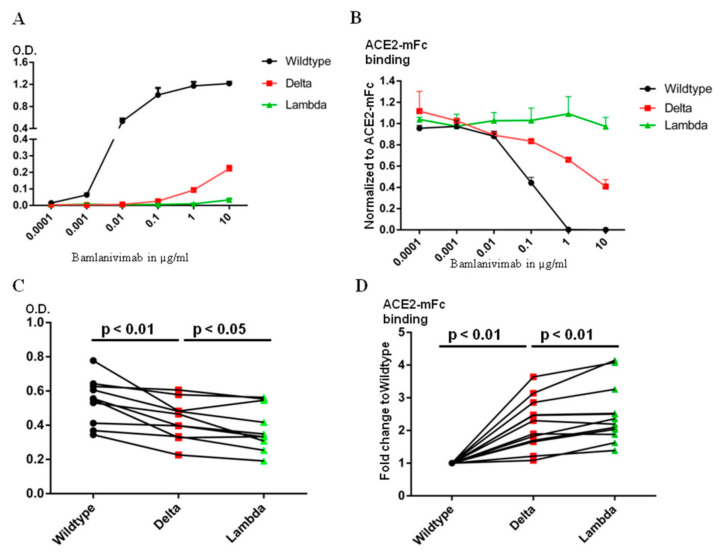
The Lambda RBD evades more Bamlanivimab and serum antibody binding from BNT162b2 mRNA vaccinated donors than Delta. (**A**). ELISA detection of Bamlanivimab binding to wild type, Delta or Lambda RBD coated plate. (**B**). ELISA detection of Bamlanivimab blocking efficacy of ACE2-mFc interaction with wild type, Delta or Lambda RBD. (**C**). BNT162b2 mRNA vaccinated sera from 10 donors were assayed on wild type, Delta or Lambda RBD coated ELISA plate to compare serum antibody evasion. The O.D. values of serum from the same donor on wild type, Delta or Lambda are linked with lines. (**D**). ELISA detection of serum antibody from 12 donors in blocking ACE2-mFc interaction with wild type, Delta or Lambda RBD. The black, red and green symbols in Figure C and D indicate signal from wild type, Delta and Lambda RBD respectively.

The epitope of Bamlanvimab overlaps with the ACE2 footprint on the RBD, and therefore binding of Bamlanivimab with the RBD blocks its interaction with ACE2 [13]. Several therapeutic monoclonal antibody drugs against the original SARS-CoV-2 virus belong to this category, including Etesivimab, Casirivimab and Imdevimab [14]. The decrease in the binding affinity to Bamlanivimab caused by the mutations in the Delta or Lambda RBD clearly affects Bamlanivimabinteraction with the Delta or Lambda RBDs. We compared Bamlanivimab binding to wild type, Delta and Lambda RBDs with an ELISA assay. Since monoclonal antibody recognizes a single epitope on the antigen, the binding of Bamlanivimab reached plateau in signal between 0.1 and 1 µg/mL (Figure 3A). In the case of 10 µg/mL Bamlanivimab binding to the Delta RBD, the signal was only at one sixth of the plateau of the wild type RBD binding due to its approximately 20-fold lower affinity. In contrast, Bamlanivimab did not bind the Lambda RBD at all, consistent with the SPR data above.

Since the mutations in the RBD region of both Delta and Lambda variants fall within or around the ACE2 footprint (Figure 1F), we used chimeric human ACE2 fused withthe mouse Fc region of the IgG ɣ2a antibody (ACE2-mFc) to evaluate the efficacy of Bamlanivimab on ACE2 blocking. Human full-length ACE2 stays as homodimer on the cell membrane [15]. The mouse Fc region not only helps ACE2 stay as homodimer, but also provides a convenient way of detection. The ACE2-mFc-based neutralization assay correlated well with pseudovirus-based neutralization assay, especially when assessing the antibodies competitiveness with ACE2 binding [16]. Both ACE2-mFc and IgG antibodies were in their dimer form. As reported here and in our earlier publication [17], the affinity of Bamlanivimab to wild type RBD was approximately 6 times higher than that of ACE2 to wild type RBD. Bamlanivimab inhibited ACE2-mFc interaction with wild type RBD in a dose-dependent manner, with 1 μg/mL of Bamlanivimab fully blocked 1 μg/mL ACE2-mFc binding to wild type RBD (Figure 3B). In contrast, the same concentration of Bamlanivimab inhibited 40% of ACE2-mFc binding to the Delta RBD, while Bamlaniviab had no blocking effect on ACE2-mFc interaction with the Lambda RBD. 

### 2.4. Serum Antibody Levels after BNT162b2 Vaccination Binds the Lambda RBD Less Efficiently Than the Delta

Bamlanivimab binds at a single epitope on the RBD and thus engages certain area of the RBD [18], but certainly not all. There are four major classes of antibody binding hotspots on the RBD [19]. Therefore, antibodies generated by vaccination should be more broadly inhibitory against SARS-CoV-2. We first used ELISA plates, coated with wild type, Delta or Lambda RBD, to compare the ability of sera from people vaccinated one week to two months after the second dose of the BNT162b2 vaccine to bind the different RBDs. The same amount of wild type, Delta or Lambda RBDs were coated on the ELISA plates as detected by HL19 monoclonal antibody targeting an epitope outside the ACE2 binding motif in the RBD region (Figure S3). Compared with the wild type RBD, the signal for serum binding to Delta and Lambda RBDs dropped to 80+/−14% and 70+/−12%, respectively (Figure 3C). We then used ACE2-mFc assay to evaluate the efficacy of serum antibodies in blocking ACE2 interaction with wild type, Delta and Lambda RBDs. We used the value of ACE2-mFc alone binding to wild type RBD as the normalization control. Consistently with serum antibody binding data, the same serum blocked ACE2-mFc binding to Lambda RBD less efficiently than the Delta RBD (Figure 3D). Thus, the Lambda RBD evaded more ACE2 blocking serum antibody binding than Delta. 

### 2.5. Serum Antibody Levels against All RBDs Fall with Time after Vaccination

It has been reported that serum antibody levels against the wild type RBD drop with time after mRNA vaccination [20]. To find out whether similar reductions occur in antibodies against the Delta and Lambda RBDs, we used serum samples collected at two weeks and six months after the second dose of the BNT162b2 mRNA vaccine from four donors. At two weeks, in these 4 individuals, there was no significant difference in serum IgG binding between wild type and Delta RBD whereas, as noted above, the level of antibodies binding the Lambda RBD was lower. At six months, the antibody against the wild type RBD had fallen to 31+/−6% of that at two weeks. In the same donors the antibody levels against the Delta and Lambda RBDs fell to 28+/−6% and 25+/−5%, respectively (Figure S4). These data show that the BNT162b2 vaccine, soon after administration, bound reasonably well against both the Delta and Lambda variants. Moreover, the waning of serum antibody levels within six months of vaccination, to about 28% of starting amounts, is independent of the RBD mutations. The waning serum antibody level after vaccination has been reported to correlate with breakthrough infections [21].

## 3. Discussion 

We dissected the effect of mutations, alone and in combination, of the Delta and Lambda variant RBD on ACE2 and Bamlanimab binding. Although both variants have two mutations in the RBD region, they have different effects depending on the location and amino acid substitutions. With Bamlanivimab as a model, our data showed that antibody affinity against the antigen correlated with its efficacy. We further analyzed serum antibody binding to wild type, Delta and Lambda RBDs, and used ACE2-mFc to identify that the Lambda RBD evades more ACE2 blocking serum antibody binding than the Delta RBD. The limitations of our research is that we did not include the full length spike bearing pseudovirus assay, as there are additional mutations outside the RBD region [8]. Research using the full length pseudovirus carrying the Delta or Lambda full length spike protein has already been carried out [22,23]. 

The wild type RBD of SAR-CoV-2 has ~6 times higher binding affinity for ACE2 than that of SARS-CoV-1 [2]. The SARS-CoV-2 full length spike protein also obtained a furin cutting site compared with the SARS-CoV-1 spike protein [24]. Compared with the wild type virus, every widely spreading variant has certain unique features for its dominance. The Alpha strain, with an N501Y mutation within the RBD, and Beta and Gamma variants bearing the same mutation with an additional E484K mutation, were dominant strains in the United Kingdom, South Africa, and Brazil, respectively. Both Delta and Lambda variants could also evade the immunity generated by vaccines [22,25]. Understanding the underlying mechanisms which trigger the higher infectivity and breakthrough infection of the variants is essential to battle the virus. We addressed the potential basis for the high infectivity of the Alpha, Beta, and Gamma variants earlier and found that the N501Y mutation within the RBD region was critical for the high infectivity of all three variant strains. While the E484K mutation within both Beta and Gamma variants, which abolished Bamlanivimab, could be the partial reason for evading the protection built up from previous infections or vaccines [17,26]. In this report, we dissected the potential factors that confer the properties of high infectivity and immune resistance within both the Delta and Lambda variant RBDs, and further suggest that the Lambda variant RBD could better evade BNT162b2 mRNA vaccine-induced humoral immunity than Delta.

From our analysis, the Delta variant RBD increases the binding affinity to ACE2 compared with the wild type strain. Meanwhile, the L452R mutation could decrease binding to the therapeutic antibody, Bamlanivimab. However, serum antibodies from subjects that were vaccinated with the BNT162b2 vaccine still bound the Delta RBD relatively well. This is consistent with the report that BNT162b2 vaccinated subjects contracting the Delta virus cleared the virus quickly [27], suggesting protection by the vaccine during the breakthrough infection [28]. Increase of ACE2 binding affinity was one reason of higher transmission rate of the Delta variant. Recent research further shows that the mutation of P681R outside of the RBD, which enhances furin cleavage, is partially responsible for the hightransmission rate of the Delta variant [29]. This supports the notion that acquisition of a furin cleavage site by SARS-CoV-2 [30], along with a much higher binding affinity to ACE2, increased the infectivity of SARS-CoV-2 [2].

Compared with the Delta variant, the Lambda RBD evades more serum antibody binding in our patient study. Although the mutations of L452Q and F490S do not enhance ACE2 binding, both mutations lead to disruption of hydrophobic patches within the RBD that are critical for the binding by several neutralizing antibodies that interfere with ACE2 interaction. The Lambda variant RBD decreased serum antibody binding as shown by our data. Two recent reports derived from the study of a pseudovirus suggest that the Lambda variant gained partial resistance to antibodies in sera from vaccinated individuals [23,31]. One of these studies proposes that mutation of T76I and L452Q lead to higher infectivity of the Lambda variant while a 7-amino-acid deletion within the N-terminal domain (outside RBD domain) accounts for immune evasion [23]. It is true that the N-terminus of the spike protein does elicit some broadly neutralizing antibodies, which neutralize the virus through mechanisms other than ACE2 blocking [32].

Interestingly, titers of antibodies dropped sharply in vaccinated people after six months, against both the original strain and variants, suggesting that the serum antibody induced by vaccines wanes over time [33,34]. This could be well expected, since serum antibodies are secreted by plasma B cells, the majority of which are short-lived [35]. In contrast, the RBD specific memory B cells stay at stable levels for at least one year after viral infection [36] or vaccination [37]. Memory B cells can provide long-term protection by rapidly proliferating into antibody secreting plasma B cells upon antigen encounter [38]. Together with the fact that T cells could also provide protection against different versions of variants [39], vaccination remains a useful tool for combating the SARS-CoV-2 virus, including potential future variants. 

## 4. Materials and Methods

### 4.1. SARS-CoV-2 RBD Plasmid Cloning and Protein Expression

SARS-CoV-2 wild type RBD (319-541aa) was cloned into the pcDNA3.1 vector with a 6-histidine tag. The Delta variant RBD with L452R and T478K mutations was created by quick change mutagenesis. The Lambda variant RBD with L452Q and F490S mutations was made in the same way. All plasmid constructs were verified by DNA sequencing. RBD proteins were expressed by transient transfection of 293F cells. RBD proteins were purified using a nickel column and further purified by a Superdex-200 Gel-filtration size column.

### 4.2. ACE2 Protein Biotinylation

Human ACE2 (1-615) with an Avi tag was cloned to pcDNA3.1 vector and expressed in 293F cells. Biotinylation of the Avi tag was carried out by birA enzyme. The biotinylated ACE2 was further purified by using a Superdex-200 Gel-filtration size column.

### 4.3. Affinity Measurement of RBD Binding to ACE2

The affinity was measured in a Biacore 3000 machine. Biotinylated ACE2 was coated on a streptavidin chip. A serial dilution of wild type or mutant RBD was injected at 20 µL/min for 1 min and dissociated for 9 min at room temperature. The affinity was calculated by BIA evaluation software.

### 4.4. Affinity Measurement of RBD Binding to Bamlanivimab

The affinity was measured in a Biacore 3000 machine. Bamlanivimab was coated on a CM5 chip using the standard amine coupling. A serial dilution of wild type or mutant RBD was injected at 20 µL/min for 1 min and dissociated for 5 min at room temperature. The bound RBD was eluted by 10 mM glycine pH 1.7. The affinity was calculated by BIA evaluation software.

### 4.5. ELISA Measurement of Serum RBD Antibody from mRNA Vaccinated Donors

Wild type, Delta or Lambda variant RBDs were coated on the same ELISA plate at 20 µg/mL overnight in cold room. The plates were washed and blocked with PBS containing 30% FBS. Human sera at two weeks after mRNA vaccine boosters and six months after boosters from four healthy donors were used. The sera were diluted at 1:100 or 1:1000. The same volume of diluted serum sample was incubated with wild type, Delta or Lambda variant RBD coated wells. The bound antibody was detected using an AP-conjugated goat-anti-human IgG Fc specific antibody (Jackson ImmunoResearch, Cat# 109-055-008). 

To exclude the possibility of different coating amount of wild type or mutant RBD to the ELISA plate, we used a RBD antibody which did not interfere with ACE2 binding as an internal control. The antibody had equal binding to wild type, Delta and Lambda RBD (Figure S3). 

### 4.6. ACE2-mFc Based Neutralization Assay

Human ACE2 (1-615) was fused to the mouse IgG ɣ2a Fc region by overlap extension PCR. The fusion DNA was cloned into pcDNA3.1 and expressed in 293F cells. The fusion protein was purified using protein A column and size exclusion column. 

For the neutralization assay, 1 μg/mL of ACE2-mFc was pre-mixed with Bamlanivimab at indicated concentration or serum at a 1:10 dilution. The same volume of mixture was then added to wild type, Delta or Lambda RBD coated ELISA plates. 1 μg/mL of ACE2-mFc alone was used as a positive control. The bound ACE2-mFc was detected using AP-conjugated anti-mouse IgG ɣ2a specific antibody (BD, Cat# 553389). 

In Bamlanivimab neutralization assays, O.D. values of ACE2-mFc alone were used as a normalization control. In serum neutralization assays, bound ACE2-mFc was first normalized to the O.D. values of ACE2-mFc alone. Due to different amounts of serum antibodies in each individual’s serum, the blocking efficacy from different samples was in a wide range. Therefore, the value of ACE2-mFc binding to the wild type RBD was used as normalization control for each individual’s serum. ACE2-mFc binding to wild type, Delta or Lambda RBD was further normalized to that of the wild type, shown as fold changes compared to the wild type. 

### 4.7. Protein Structure Modeling

The crystal structures of SARS-CoV-2 wild type or Delta variant RBD bound with ACE2 (PDB code: 6M0J for wild type RBD, 7W9I for Delta RBD) were selected for structural analysis. The Lambda variant mutant (L452Q and_F490S) structure was prepared using the Coot software. All protein structural figures were prepared using the PyMOL software.

### 4.8. Human Serum Sample

The use of human PBMC samples from donors before and after BNT162b2 mRNA vaccination was approved by the National Jewish Health COVID-19 Research coordination committee. All the donors had received two doses of 30 μg BNT162b2 COVID-19 mRNA vaccine. The ages of the donors are ranged from 30 to 70 years old. The human blood samples were collected by the biobank of National Jewish Health and all the participants in the study provided written consent. All the human samples were de-identified.

### 4.9. Statistical Analysis

Paired T tests were used to analyze the *p* value of serum antibody binding and blocking of ACE2-mFc to wild type, Delta or Lambda RBD.

## Data Availability

The data presented in this study are available on request from the corresponding author.

## Supplementary Materials

The following supporting information can be downloaded at: https://www.mdpi.com/article/10.3390/ijms231911325/s1.
