# Supplementary material for: The Receptor Binding Domain of SARS-CoV-2 Lambda Variant Has a Better Chance Than the Delta Variant in Evading BNT162b2 COVID-19 mRNA Vaccine-Induced Humoral Immunity
Source: Int J Mol Sci. 2022 Sep 26;23(19):11325. doi: 10.3390/ijms231911325 (PMC9569855; doi:10.3390/ijms231911325)
Supplement: Supplementary file 1 [file ijms-23-11325-s001.zip › ijms-1848897-supplementary.pdf]

Supplementary Figure

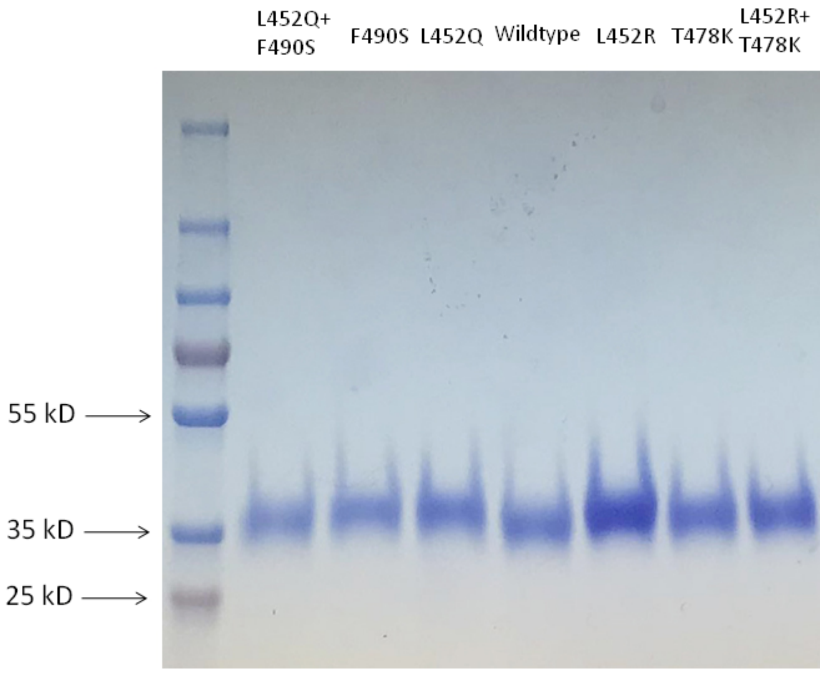

Figure S1. SDS-PAGE gel image of purified SARS-CoV-2 RBD with indicated mutations.

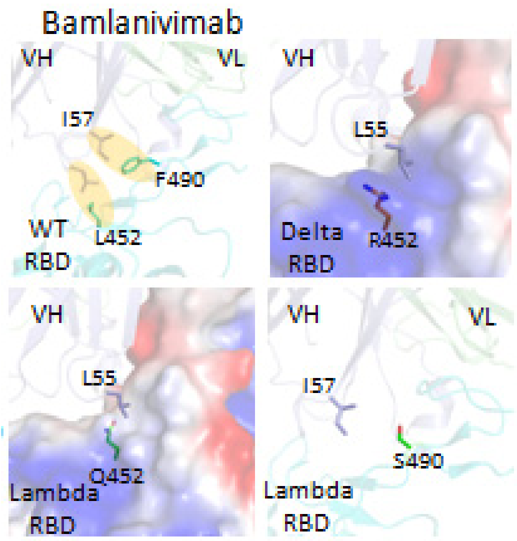

Figure S2. Protein modeling between Bamlanivimab and wildtype, Delta or Lambda RBDs. The interaction between Leucine 452 or Phenylalanine 490 of wildtype RBD and Leucine 55 or Isoleucine 57, respectively, of Bamlanivimab (Upper left). L452R (Upper right) or L452Q (Lower left) would interrupt the hydrophobic interactions and lead to the decrease of Bamlanivimab affinity. F490S (Lower right) would also interrupt the orginal hydrophobic interaction. 7KMG (PDB ID) was used for modeling.

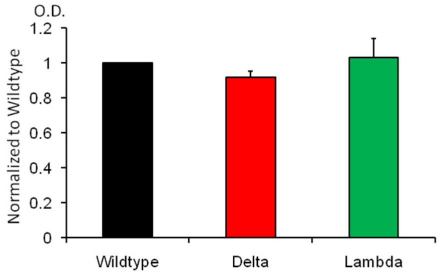

Figure S3. HL19, a mouse monoclonal antibody, which doesn't interfere with ACE2 binding, binds wildtype, Delta and Lambda RBD equally well.

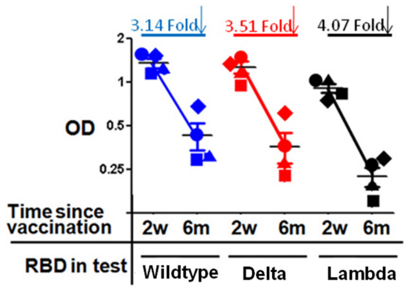

Figure S4. Serum antibody is waning againt both wildtype and variant RBDs after second dose of BNT162b2 mRNA vaccination. Serum collected from 4 donors at 2 weeks and 6 months after second dose of BNT162b2 mRNA vaccine was assayed on wildtype, Delta or Lambda RBD coated ELISA plate to compare the decay of serum antibody against different RBDs. The same label in wildtype, Delta or Lambda group indicates serum from the same donor.
